# Impact of the 2021 European Resuscitation Council-European Society of Intensive Care Medicine (ERC-ESICM) Guidelines on Neuroprognostication Practices After Cardiac Arrest: A Five-Year Cohort Study

**DOI:** 10.7759/cureus.102685

**Published:** 2026-01-31

**Authors:** Debora Lopes, Diana Martins, José Manuel Pereira, José-Artur Paiva

**Affiliations:** 1 Intensive Care Medicine, Unidade Local de Saúde do Médio Tejo, Abrantes, PRT; 2 Intensive Care Medicine, Unidade Local de Saude de São João, Porto, PRT; 3 Medicine, Faculty of Medicine, University of Porto, Porto, PRT

**Keywords:** cardiac arrest, icu survival, neuroprognostication, resuscitation, withdrawal of life-sustaining measures

## Abstract

Background: Accurate neuroprognostication after cardiac arrest (CA) is essential to avoid premature withdrawal of life-sustaining measures (WLSM). The 2021 European Resuscitation Council/European Society of Intensive Care Medicine (ERC-ESICM) guidelines strengthened multimodal prognostication, but real adherence and its relationship with clinical outcomes remain insufficiently evaluated.

Methods: We conducted a retrospective cohort study of adult CA patients admitted to the Intensive Care Unit (ICU) between 2018 and 2022. Adherence to seven guideline-based neuroprognostication parameters was quantified through a composite adherence score (0-7). Cohorts were compared before (pre-guidelines) and after (post-guidelines) March 2021. Temporal trends were examined from 2018 to 2022. Associations between adherence and four clinical outcomes (WLSM ≥ 72 hours, overall WLSM, ICU mortality, and six-month mortality) were assessed using Mann-Whitney U tests and rank-biserial correlations.

Results: A total of 301 patients were included (pre-guidelines: 200; post-guidelines: 101). Neuron-specific enolase (NSE) measurement increased markedly after guideline publication (1% vs. 34.6%, p < 0.01), whereas adherence to somatosensory evoked potentials (SSEP), electroencephalogram (EEG), neuroimaging, neuroprognostication ≥ 72 hours, and deferred WLSM ≥ 72 hours did not differ significantly between cohorts. The composite adherence score increased from 3 (1-4) to 4 (1-5) (p = 0.056). Across 2018-2022, mean adherence demonstrated a gradual upward trend, with the sharpest rise after 2021. Individual parameter trends were heterogeneous, with significant post-guideline increases only in NSE use. Higher adherence scores were significantly associated with WLSM ≥ 72 hours, overall WLSM, and lower ICU and six-month mortality (all p < 0.05). Rank-biserial correlations showed moderate positive associations with WLSM variables and moderate negative associations with mortality outcomes.

Conclusions: Implementation of the 2021 ERC-ESICM guidelines was associated with increased overall adherence, primarily driven by NSE measurement. Higher adherence correlated with more guideline-concordant timing of WLSM and improved survival outcomes. Strengthening multimodal prognostication practice may further reduce variability and prevent premature care limitation.

## Introduction

Cardiac arrest (CA) is a major global public health challenge and a leading cause of premature death. Each year, an estimated 343,500 people experience out-of-hospital CA (OHCA) and 80,000 to 100,000 people experience in-hospital CA (IHCA) in Europe [1,2]. Although the total number of years of potential life lost (YPLL) due to CA in Europe is not precisely quantified, data from the United States indicate that CA accounts for approximately 3.3 million YPLL annually, and the European burden is likely comparable [3].

Following successful resuscitation, approximately 80% of patients remain comatose, and nearly two-thirds ultimately die as a consequence of hypoxic-ischemic brain injury. Importantly, only a minority of these deaths result from irreversible neurological damage such as brain death; most occur following withdrawal of life-sustaining treatment (WLST) after prognostication of poor neurological outcome [4].

To minimize premature or inaccurate WLST in patients with potential for neurological recovery, the European Resuscitation Council (ERC) first introduced a dedicated post-resuscitation care section within the 2010 advanced life support guidelines, subsequently updating it every five years [5]. The 2021 ERC/European Society of Intensive Care Medicine (ERC-ESICM) guidelines refined this approach, emphasizing a strictly multimodal prognostication algorithm, standardized timing of assessment, broadened entry criteria, and the inclusion of good outcome predictors [6]. These modifications increased the specificity of poor-outcome prediction, thereby reducing premature WLST; however, the impact of guideline implementation on survival and neurological outcomes remains insufficiently studied, and a substantial proportion of patients continue to be classified as having an indeterminate prognosis, results being limited, for example, by the high rate of withdrawal of life-sustaining therapies [7].

Efforts have been employed, such as a large pooled analysis of four multicenter cohorts (TTM, TTM2, KORHN, and ProNeCA), showing that the 2021 guideline criteria have good specificity for poor outcome while reducing the proportion of patients with indeterminate prognosis when favorable predictors are included, thus decreasing prognostic uncertainty [8]. Additionally, prospective data from a 28-center European cohort demonstrated that the guideline algorithm achieved a 100% positive predictive value for poor neurological outcome; however, half of the patients remained in the indeterminate category [9]. Despite these advances, real-world adherence to neuroprognostication recommendations varies widely across institutions and may influence prognostic accuracy, WLST practices, and ultimately patient outcomes, namely, survival and neurological recovery [10]. Therefore, important gaps remain: the focus on the predictive performance of guideline criteria rather than on how guideline implementation influences decisions such as WLST or survival in real-world practice. Our study addresses this gap by evaluating the association between guideline adherence and key clinical outcomes, thereby providing novel insight into the practical impact of guideline implementation beyond prognostic accuracy alone. Assessing the degree to which clinical practice adheres to guideline recommendations-especially following the 2021 update [6]-is crucial in order to optimize care pathways, avoid self-fulfilling prognoses due to premature decisions, and prevent delayed identification of futility that can result in unnecessary suffering and increased resource utilization.

In this context, the study aimed to evaluate the implementation of the 2021 ERC-ESICM neuroprognostication guidelines in comatose post-CA patients admitted to the Intensive Care Unit (ICU) [6]. Specifically, adherence to key guideline-recommended prognostic parameters was assessed using a predefined composite adherence score, compared adherence before and after guideline publication, examined temporal trends, and explored associations between adherence and clinically relevant outcomes. The primary objective was descriptive, assessing adherence to the 2021 ERC-ESICM neuroprognostication guidelines, while secondary objectives were hypothesis-driven, evaluating temporal trends and associations between adherence and clinical outcomes.

## Materials and methods

Study design and setting

A retrospective observational cohort study was conducted of consecutive patients admitted to a single ICU at a tertiary referral center, involving medical and surgical care patients after successful resuscitation from OHCA and IHCA between January 2018 and December 2022. Throughout the study period, patients were managed according to a standardized post-CA care protocol, including targeted temperature management, hemodynamic optimization, ventilatory support, and guideline-based neuroprognostication, which remained consistent over time. To assess the impact of the 2021 ERC-ESICM guidelines for post-resuscitation care [6], the cohort was divided into two groups: a pre-guideline group (January 2018-March 2021) and a post-guideline group (April 2021-December 2022).

The study is under the framework of the Strengthening the Reporting of Observational Studies in Epidemiology (STROBE) 2014 guideline [11]. The institutional ethics committee reviewed and approved the study and waived the requirement for patient consent. All the procedures followed the ethical standards and complied with the Helsinki Declaration. Data processing was conducted anonymously, adhering to the principles outlined in the European General Data Protection Regulation.

Eligibility criteria

Inclusion criteria encompassed adults aged over 18 years, men or non-pregnant women, and patients who remained comatose after return of spontaneous circulation following IHCA or OHCA and were admitted to the ICU for post-resuscitation care. To assess guideline adherence, only patients comatose for at least 24 hours following ICU admission were included in the analyses. The 24-hour threshold was chosen to allow sufficient time for initial stabilization and clinical assessment, consistent with prior post-CA prognostication studies [12,13]. Patients considered potential organ donors upon admission or undergoing extracorporeal cardiopulmonary resuscitation (eCPR) were excluded. Patients transferred from another ICU at any time or transferred to another ICU within the first 24 hours were also excluded. Patients who regained consciousness within 24 hours were not captured in the adherence analyses, which may have slightly overestimated adherence among the comatose population.

Objectives and endpoints

The primary objective was to evaluate the impact of the guideline implementation on WLST, ICU survival, and favorable outcome at ICU discharge. Secondary objectives included describing the trend in adherence to guideline recommendations. Primary endpoints included timing and rate of WLST, survival to ICU discharge, and neurological outcome at ICU discharge. Neurological outcome was assessed with the Cerebral Performance Category (CPC), with a score of 1-2 defined as a good neurological outcome and a CPC score of 3-5 as a poor outcome. Guideline adherence was assessed based on the execution of the recommended procedures/interventions during the ICU stay.

To quantify adherence to neuroprognostication recommendations, we developed a composite adherence score based on seven predefined guideline-derived parameters. By design, all parameters were weighted equally, with each fulfilled parameter contributing one point, yielding a total score ranging from 0 to 7, with higher scores reflecting greater adherence. The parameters included (1) measurement of neuron-specific enolase (NSE), (2) somatosensory evoked potentials (SSEP), (3) neuroprognostication performed ≥72 hours after return of spontaneous circulation, (4) withdrawal of life-sustaining measures (WLSM) deferred to ≥72 hours, (5) electroencephalogram (EEG) assessment, (6) neuroimaging evaluation, and (7) use of at least two prognostic modalities, in line with guideline recommendations.

EEG recordings were interpreted by experienced neurophysiologists according to standardized post-CA criteria, and eligible patterns of classification were Suppression, Burst-Suppression, Seizure Activity, or Inconclusive. NSE concentrations were measured using a commercially available immunoassay at specific timings-24 hours, 48 hours, or 72 hours, depending on clinical judgment and availability.

The adherence score was analyzed as a continuous variable for comparisons between the pre-guideline and post-guideline cohorts. Each individual parameter was also assessed independently as a binary variable (adherent vs. non-adherent). Temporal patterns in adherence were evaluated by calculating yearly mean adherence scores and yearly proportions of adherence for each parameter from 2018 to 2022.

To explore whether adherence was associated with clinical outcomes, we examined the relationship between the final adherence score and four predefined variables: WLSM ≥ 72 hours, WLSM (any), ICU mortality, and six-month mortality. Because the adherence score was non-normally distributed, comparisons of adherence scores between outcome groups (“yes” vs. “no”) were performed using the Mann-Whitney U test for two-group comparisons and Kruskal-Wallis tests for comparisons involving more than two groups. When Kruskal-Wallis tests indicated statistically significant differences, post hoc pairwise comparisons were performed with Bonferroni adjustment to account for multiple testing. Adjusted results are reported when they differ from the primary (unadjusted) analyses. In addition, rank-biserial correlations were calculated to quantify the strength and direction of association between the continuous adherence score and each binary outcome.

Statistical analysis

For statistical analysis, SPSS (IBM Corp., released 2022; IBM SPSS Statistics for Windows, version 29.0, Armonk, NY, USA) was used. Data were presented as n (%) or reported using mean and standard deviation or median and interquartile range (IQR), depending on normality assumptions. The Mann-Whitney U test was used for continuous non-normally distributed variables, which were described as median (IQR), and the Chi-squared test and Fisher’s exact test for categorical variables; p-values are two-tailed. Statistically significant results were set at a p < 0.05 level.

Clinical setting

The institutional standard of practice for neuroprognostication evolved during the study period, transitioning from the 2015 guideline recommendations before 2021 to the 2021 ERC-ESICM guidelines. Before 2021, neuroprognostication was performed typically based on clinical examination, early EEG, and neuroimaging findings. Use of additional modalities such as SSEP or NSE measurements was not standardized and depended on clinical judgment and test availability. After the publication of the 2021 ERC-ESICM guidelines [6], a structured institutional protocol for post-CA care was implemented, with routine use of multimodal prognostication, integrating neurological examination (motor response ≤ 3 at 72 hours), continuous or intermittent EEG monitoring (>24 hours), bilateral SSEP assessment (presence or absence of N20 responses), serial serum NSE measurements at 48 and 72 hours, and brain imaging (computed tomography (CT) and/or magnetic resonance imaging (MRI)) to detect diffuse anoxic injury.

## Results

Study population

During the study period, 347 CA survivors were admitted to the ICU. A total of 301 patients met the inclusion criteria; 46 were excluded due to admission for organ donation following brain death or cannulation for veno-arterial extracorporeal membrane oxygenation (ECMO) under the eCPR protocol. Patients were divided into two cohorts: pre-guideline period (January 2018-March 2021): 200 patients (66.4%) and post-guideline period (April 2021-December 2022): 101 patients (33.6%).

Baseline characteristics and disease severity

Baseline characteristics are summarized in Table 1. The overall median age was 67 years, with patients in the post-guideline cohort slightly younger (68 vs. 63 years; p = 0.046). This difference likely reflects indication or selection bias rather than a causal effect of guideline implementation, so given the modest magnitude of the difference and its likely non-causal nature, age was not included as a covariate in outcome analyses. The five-year difference, while statistically significant, is modest, and its clinical relevance is uncertain. Sex distribution was similar, with approximately 71% men in both groups. Most comorbidities were comparable between groups, with smoking, hypertension, and dyslipidemia being the most common ones. Malignant disease was more frequent in the pre-guideline cohort (12% vs. 1%; p = 0.001), and end-stage liver disease showed a marginally significant difference (7.5% vs. 2%; p = 0.050). CA characteristics were broadly similar. “Out-of-hospital at home” was the most common CA location in both cohorts. Initial rhythm distribution varied descriptively (more ventricular fibrillation (VF) in pre-guidelines; more pulseless electrical activity (PEA) in post-guidelines), though without statistical significance. No-flow time was longer in the post-guideline cohort (2.65 vs. 5.10 minutes; p = 0.010). Cardiac etiology remained the most common overall, but the proportion differed between groups, excluding the unknown causes (p = 0.013). Admission severity scores were largely comparable, except for a higher Sequential Organ Failure Assessment (SOFA) score at 24 hours in the post-guideline group (9 vs. 10; p = 0.046). Lactate, arterial pH, and intubation rates showed no significant differences.

**Table 1:** Baseline patient and cardiac arrest characteristics and disease severity ^1^Mann-Whitney U test ^2^Chi-squared test ^3^Chi-squared test excluding unknown causes BMI: body mass index; COPD: chronic obstructive pulmonary disease; SAPS II: Simplified Acute Physiology Score II; SOFA: Sequential Organ Failure Assessment; IQR: interquartile range; ROSC: return of spontaneous circulation

	All (n = 301)	Pre-guidelines (n = 200)	Post-guidelines (n = 101)	Test value	p-value
Age, years-median (IQR)	67 (55-76)	68 (55-77)	63 (55-74)	Z = -1.998	0.046^1^
Male, n (%)	213 (70.76%)	142 (71%)	71 (70.30%)	χ² = 0.016	0.899^2^
Comorbidities, n (%)					
Coronary artery disease	98 (32.56%)	66 (33%)	32 (31.68%)	χ² = 0.053	0.818^2^
Congestive heart disease	77 (25.58%)	50 (25%)	27 (26.73%)	χ² = 0.106	0.745^2^
Smoking	127 (42.19%)	84 (42%)	43 (42.57%)	χ² = 0.009	0.924^2^
Alcohol use disorder	52 (17.28%)	37 (18.50%)	15 (14.85%)	χ² = 0.625	0.429^2^
Dyslipidemia	149 (49.50%)	105 (52.50%)	44 (43.56%)	χ² = 2.144	0.143^2^
Hypertension	174 (57.81%)	114 (57%)	60 (59.41%)	χ² = 0.159	0.690^2^
Diabetes mellitus	74 (24.58%)	47 (23.50%)	27 (26.73%)	χ² = 0.378	0.539^2^
Obesity (BMI > 30 kg/m²)	66 (21.93%)	39 (19.50%)	27 (26.73%)	χ² = 2.051	0.152^2^
Chronic kidney disease	44 (14.62%)	31 (15.50%)	13 (12.87%)	χ² = 0.372	0.542^2^
End-stage liver disease	17 (5.65%)	15 (7.50%)	2 (1.98%)	χ² = 3.837	0.050^2^
COPD	39 (12.96%)	23 (11.50%)	16 (15.84%)	χ² = 1.122	0.290^2^
Malignant disease	25 (8.31%)	24 (12%)	1 (0.99%)	NA	0.001^3^
Cardiac arrest location, n (%)					
In-hospital-monitored ward	66 (21.93%)	44 (22%)	22 (21.78%)	χ² = 0.175	0.676^2^
In-hospital-non-monitored ward	70 (23.26%)	49 (24.50%)	21 (20.79%)		
Out-of-hospital at home	91 (30.23%)	60 (30%)	31 (30.69%)	χ² = 0.105	0.746^2^
Out-of-hospital in public space	74 (24.58%)	47 (23.50%)	27 (26.73%)		
Initial rhythm, n (%)					
Ventricular fibrillation	94 (21.23%)	62 (31%)	32 (31.68%)	χ² = 0.015	0.904^2^
Pulseless vent. tachycardia	35 (11.63%)	26 (13%)	9 (8.91%)	χ² = 1.092	0.296^2^
Pulseless electrical activity	95 (31.56%)	57 (28.50%)	38 (37.62%)	χ² = 2.586	0.108^2^
Asystole	66 (21.93%)	46 (23%)	20 (19.80%)	χ² = 0.401	0.527^2^
Unknown	11 (3.65%)	9 (4.50%)	2 (1.98%)	χ² = 1.210	0.271^2^
Time to ROSC (minutes)					
No-flow	3.46 (6.05)	2.65 (4.43)	5.10 (8.16)	Z = 2.567	0.010^1^
Low-flow	14.37 (12.51)	14.94 (13.02)	13.24 (11.41)	Z = -0.667	0.506^1^
Cardiac arrest cause, n (%)					
Cardiac cause	166 (55.15%)	115 (57.50%)	51 (50.50%)	χ² = 8.673	0.013^3^
Non-cardiac cause	86 (28.57%)	47 (23.50%)	39 (38.61%)		
Unknown	49 (16.28%)	38 (19%)	11 (10.89%)		
Disease severity					
SAPS II-median (IQR)	61 (46-71)	60 (45-71)	62.5 (47-71)	Z = 0.906	0.365^1^
SOFA at 24 h-median (IQR)	10 (7-12)	9 (7-11)	10 (8-12)	Z = 1.992	0.046^1^
Lactic acid (mmol/L) (IQR)	6.10 (3.8-9.14)	6.05 (3.98-9.18)	6.62 (3.5-9.10)	Z = 0.008	0.994^1^
pH-median (IQR)	7.22 (7.05-7.31)	7.22 (7.03-7.30)	7.22 (7.03-7.32)	Z = 0.332	0.740^1^
Endotracheal intubation (IQR)	3 (2-6)	3 (2-6)	4 (2-6)	Z = 1.162	0.245^1^

Neuroprognostication practice and results

Neuroprognostication practices and results are summarized in Table 2. The proportion of patients with a motor score ≤ 3 at 72 hours-triggering formal neuroprognostication-was similar between cohorts (48% vs. 50.5%; p = 0.588). After guideline publication, NSE measurement increased markedly, resulting in significantly more patients identified with NSE > 60 µg/L (p < 0.001). Bilateral absence of the SSEP N20 response occurred in 24 patients overall, with no significant differences between cohorts. Timing of SSEP assessments was similar (mean 4.2 days; p = 0.824). Regarding EEG, while the proportion of patients performing EEG did not differ significantly between cohorts, certain EEG patterns did. Burst-suppression and electrographic seizure activity were more frequent in the post-guideline cohort (p = 0.035 and p = 0.025, respectively). Suppression pattern differences approached significance (p = 0.055). EEG timing was comparable (mean 3.2 days; p = 0.371). Neuroimaging findings compatible with anoxic brain injury (generalized brain edema, reduced gray/white ratio on CT, or diffusion restriction on MRI) did not differ significantly between groups, and imaging timing was similar (mean three days; p = 0.266). Clinical signs associated with poor prognosis-including myoclonus and fever at 72 hours-occurred with similar frequency in both periods.

**Table 2:** Neuroprognostication after resuscitation from cardiac arrest ^1^Chi-squared test ^2^Fisher’s exact test ^3^Mann-Whitney U test M: Glasgow Coma Score Motor Response; NSE: neuron-specific enolase; SSEP: somatosensory evoked potentials; EEG: electroencephalography; CT: computed tomography; MRI: magnetic resonance imaging; SD: standard deviation

	All (n = 301)	Pre-guidelines (n = 200)	Post-guidelines (n = 101)	Test value	p-value
M ≤ 3 at 72 h without confounders (n/%)	147 (48.84%)	96 (48%)	51 (50.50%)	χ² = 0.294	0.588^1^
NSE > 60 μg/L at 48 h (n/%)	13 (48.15%)	0 (0%)	13 (50%)	NA	<0.001^2^
NSE > 60 μg/L at 72 h (n/%)	14 (60.87%)	1 (100%)	13 (50%)	NA	<0.001^2^
SSEP-N20 absent bilaterally (n/%)	24 (48.98%)	12 (44%)	12 (54.55%)	χ² = 3.163	0.075^1^
SSEP-N20 present bilaterally (n/%)	24 (48.98%)	14 (51.85%)	10 (45.45%)	χ² = 0.770	0.380^1^
SSEP inconclusive (n/%)	1 (2.04%)	1 (3.70%)	0 (0%)	NA	0.066^2^
Date of SSEP assessment (mean ± SD)	4.23 ± 1.79	4.22 ± 1.76	4.24 ± 1.87	Z = -0.222	0.824^3^
EEG at >24 h (n/%)	161 (53.49%)	108 (54%)	53 (52.48%)	χ² = 0.063	0.802^1^
Suppression	90 (55.90%)	67 (62.04%)	23 (43.40%)	χ² = 3.685	0.055^1^
Burst-suppression	27 (16.77%)	13 (12.04%)	14 (26.42%)	χ² = 4.454	0.035^1^
Seizure activity	31 (19.25%)	15 (13.89%)	16 (30.19%)	χ² = 5.055	0.025^1^
Inconclusive	13 (8.07%)	13 (12.04%)	0 (0%)	NA	0.006^2^
Date of EEG assessment (mean ± SD)	3.19 ± 1.94	3.18 ± 1.54	3.23 ± 2.58	Z = -0.895	0.371^3^
Status myoclonus ≤ 72 h (n/%)	64 (21.26%)	40 (20%)	24 (23.76%)	χ² = 1.043	0.594^1^
Anoxic brain injury CT/MRI (n/%)					
Generalized brain edema	56 (28.79%)	33 (24.81%)	23 (35.38%)	χ² = 1.743	0.187^1^
Reduction gray/white matter ratio	38 (18.69%)	30 (22.56%)	8 (12.31%)	χ² = 3.049	0.081^1^
Diffusion restriction on MRI	5 (2.53%)	3 (2.26%)	2 (3.08%)	χ² = 0.095	0.758^1^
Inconclusive	99 (50%)	67 (50.38%)	32 (49.23%)	χ² = 0.100	0.751^1^
Date of CT/MRI assessment (mean ± SD)	3.11 ± 2.01	2.81 ± 1.74	3.70 ± 3.45	Z = 1.112	0.266^3^
Fever ≤ 72 h (n/%)	75 (24.92%)	51 (25.50%)	24 (23.76%)	χ² = 0.108	0.742^1^

Clinical outcomes

Clinical outcomes are presented in Table 3. Overall ICU mortality was 46.2%, with no significant differences between the pre- and post-guideline cohorts (p = 0.688). Six-month mortality was also similar (p = 0.219). WLSM occurred in 39% of patients in the pre-guideline period and 32.7% in the post-guideline period (p = 0.446), with no difference in the proportion of WLSM performed ≥72 hours. Death from other causes (non-neurological) and CPC score were comparable between groups. Favorable outcomes (CPC 1-2) occurred in 48% of pre-guideline and 43.6% of post-guideline patients (p = 0.466).

**Table 3:** Patient outcomes ^1^Mann-Whitney U test ^2^Chi-squared test ^3^Fisher’s exact test ICU: Intensive Care Unit; WLSM: withdrawal of life-sustaining measures; CPC: Cerebral Performance Category; IQR: interquartile range

	All (n = 301)	Pre-guidelines (n = 200)	Post-guidelines (n = 101)	Test value	p-value
ICU length of stay (median + IQR)	5 (3-8)	5 (3-8)	5 (3-9)	Z = 1.066	0.287^1^
ICU mortality n (%)	139 (46.18%)	94 (47%)	45 (44.55%)	χ² = 0.161	0.688^2^
WLSM (n/%)	111 (36.88%)	78 (39%)	33 (32.67%)	χ² = 1.154	0.283^2^
WLSM < 72 h (n/%)	57 (18.94%)	41 (20.50%)	17 (16.83%)	χ² = 0.581	0.446^2^
Death from other causes (n/%)	28 (9.30%)	17 (8.50%)	11 (10.89%)	χ² = 0.944	0.624^2^
CPC at discharge n (%)					
CPC 1-2	141 (46.84%)	96 (48%)	44 (43.58%)	χ² = 0.531	0.466^2^
CPC 3-5	160 (53.16%)	104 (52%)	57 (56.44%)		
Mortality at 6 months (n/%)	161 (53.49%)	112 (56%)	49 (48.51%)	χ² = 1.511	0.219^2^

Adherence to guidelines

Adherence to individual neuroprognostication parameters and the final composite score, as explained in Methods, are summarized in Table 4 for the pre-guideline cohort and for the post-guideline cohort.

**Table 4:** Adherence to neuroprognostication parameters before and after implementation of the 2021 guidelines ^1^Fisher’s exact test ^2^Chi-squared test ^3^Mann-Whitney U test NSE: neuron-specific enolase; SSEP: somatosensory evoked potentials; WLSM: withdrawal of life-sustaining measures; EEG: electroencephalography; IQR: interquartile range

Parameter	Pre-guidelines n (%)	Post-guidelines n (%)	Test value	p-value
NSE	2 (1%)	35 (34.65%)	NA	<0.01^1^
SSEP	27 (13.50%)	22 (21.78%)	χ² = 3.378	0.066^2^
Neuroprognostication ≥ 72 h	44 (22%)	17 (16.83%)	χ² = 1.109	0.29^2^
WLSM ≥ 72h	159 (79.50%)	85 (84.16%)	χ² = 0.949	0.33^2^
EEG	106 (53%)	53 (52.48%)	χ² = 0.007	0.93^2^
Imaging	128 (64%)	63 (62.38%)	χ² = 0.076	0.78^2^
≥2 parameters	122 (61%)	65 (64.36%)	χ² = 0.321	0.57^2^
Final adherence score (median (IQR))	3 (1.0-4.0)	4 (1.0-5.0)	Z = -1.84643	0.056^3^

**Figure 1:**
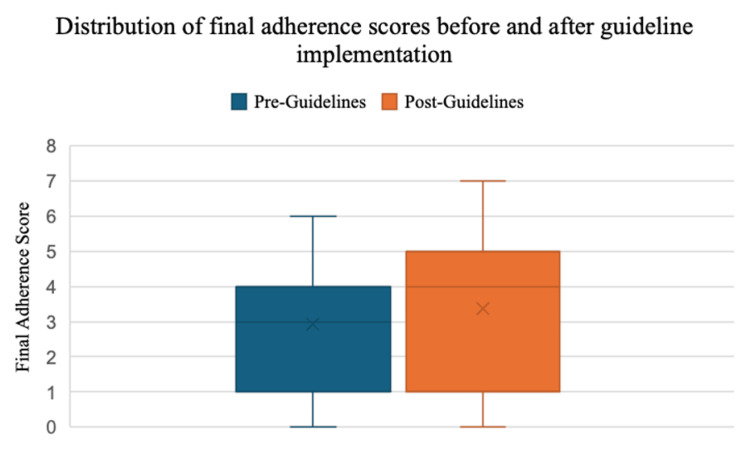
Distribution of final adherence scores before and after guideline implementation

Overall adherence to most parameters was similar between cohorts, with the exception of NSE measurement, which increased substantially in the post-guideline period (1% vs. 34.6%, p < 0.01). No statistically significant differences were observed for SSEP, EEG, neuroimaging, timing of neuroprognostication ≥ 72 hours, or deferral of WLSM ≥ 72 hours. The proportion of patients in whom ≥2 prognostic modalities were used remained similar between cohorts. Regarding the final adherence score, it increased from 3 (1.0-4.0) to 4 (1.0-5.0), with a marginally significant difference. Figure 1 highlights the distribution of both cohorts.

Evolution of adherence over time

**Figure 2:**
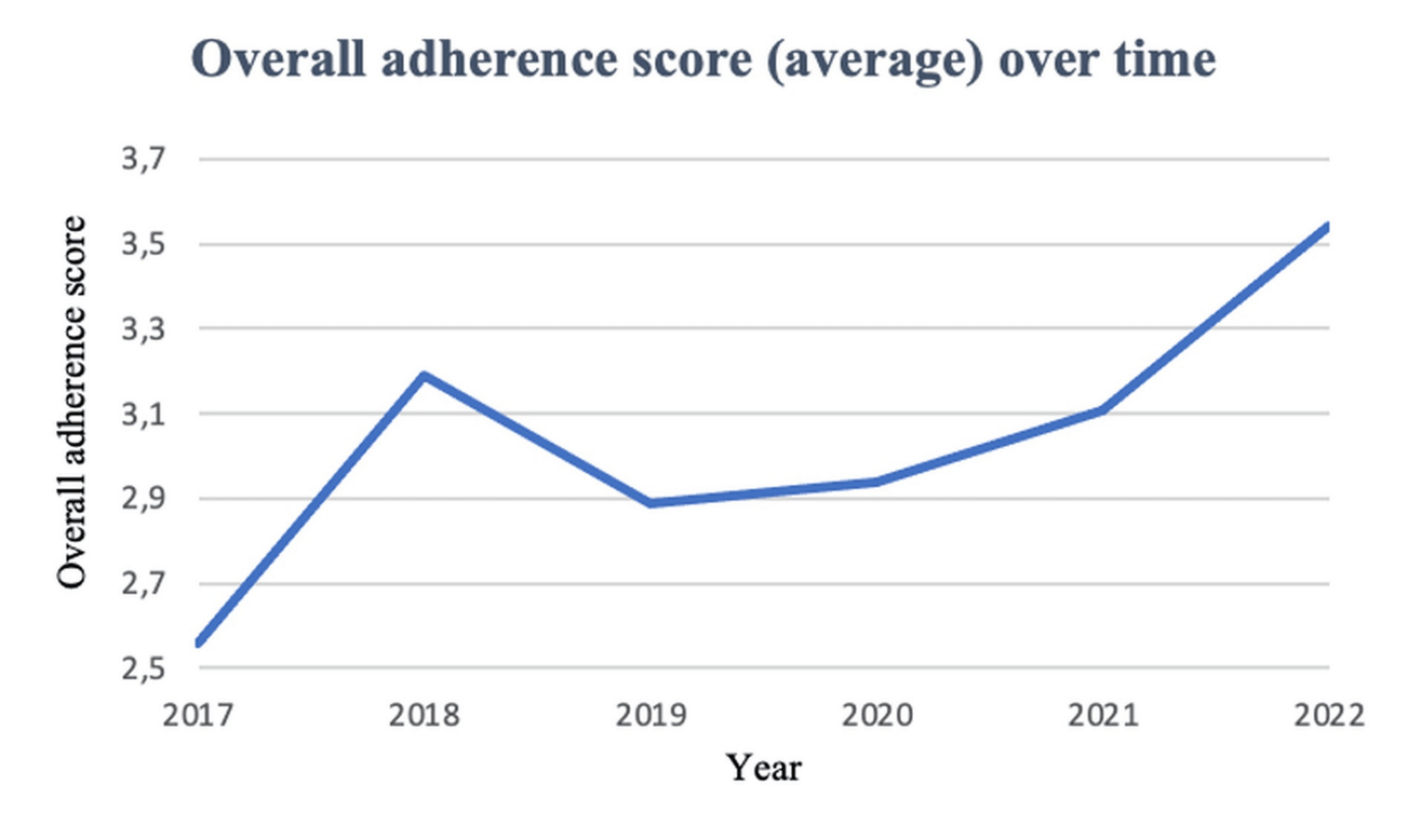
Overall adherence score (average) over time

**Figure 3:**
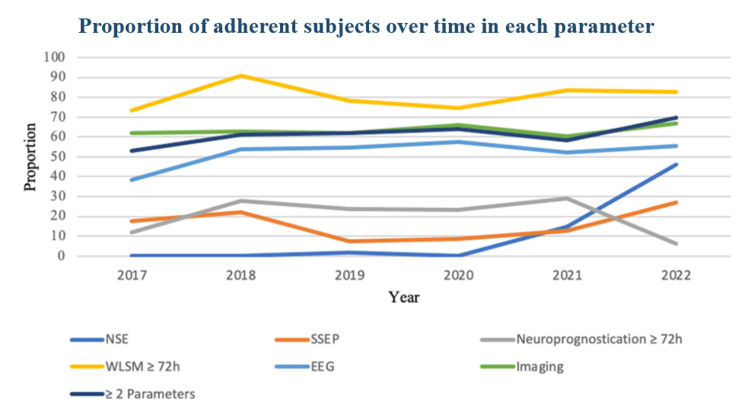
Proportion of adherent subjects over time in each parameter NSE: neuron-specific enolase; WLSM: withdrawal of life-sustaining measures; SSEP: somatosensory evoked potentials; EEG: electroencephalography

Annual mean adherence scores from 2018 to 2022 are displayed in Figure 2. There was a gradual upward trend over the study period, with the highest adherence observed after publication of the 2021 guidelines [6]. The annual proportion of adherence to each individual parameter is presented in Figure 3, showing heterogeneous evolution across predictors. NSE use increased markedly after 2021, whereas EEG and imaging remained consistently high across the study period.

Associations and correlations between the final adherence score and clinical outcomes

**Figure 4:**
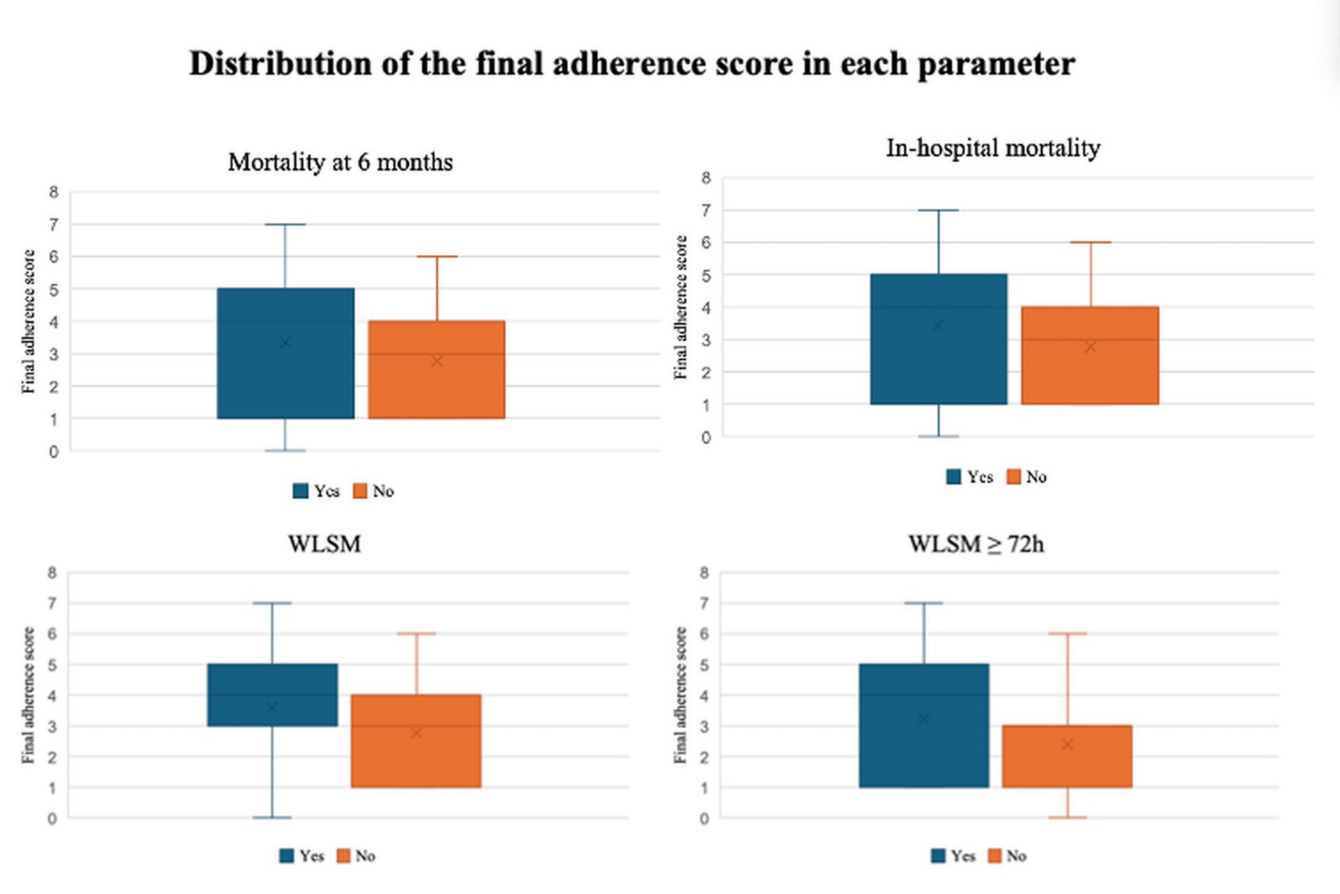
Distribution of the final adherence score in each parameter WLSM: withdrawal of life-sustaining measures

The composite adherence score was compared between patients with and without each clinical outcome. Higher adherence scores were significantly associated with both WLSM ≥ 72 hours and overall WLSM, and both ICU mortality and six-month mortality (Table 5 and Figure 4).

**Table 5:** Comparison of final adherence scores by clinical outcome ^1^Mann-Whitney U test WLSM: withdrawal of life-sustaining measures; ICU: Intensive Care Unit; IQR: interquartile range

Parameter	Yes (n), median (IQR)	No (n), median (IQR)	Test value	p-value
WLSM	111, 4 (3-5)	190, 3 (1-4)	Z = -3.32173	0.0009^1^
WLSM ≥ 72 h	244, 4 (1-5)	57, 3 (1-3)	Z = 3.37461	0.00076^1^
ICU mortality	139, 4 (1-5)	162, 3 (1-4)	Z = -2.80155	0.00512^1^
Mortality at 6 months	161, 4 (1-5)	140, 3 (1-4)	Z = 2.3149	0.02088^1^

To further quantify these relationships, rank-biserial correlations were calculated (Table 6). Adherence showed a moderate positive and significant association with WLSM and WLSM ≥ 72 hours, as well as negative significant correlations with mortality outcomes.

**Table 6:** Correlation between final adherence score and clinical outcomes ^1^Rank-biserial correlation (0 = WLSM not performed, 1 = WLSM performed) ^2^Rank-biserial correlation (0 = WLSM < 72 h, 1 = WLSM ≥ 72 h or not performed at all) ^3^Rank-biserial correlation (0 = died in hospital, 1 = discharged) ^4^Rank-biserial correlation (0 = died within 6 months, 1 = survived at least 6 months) WLSM: withdrawal of life-sustaining measures; ICU: Intensive Care Unit

Parameter	r-value (p-value)
WLSM	0.21724 (0.00015)^1^
WLSM ≥ 72 h	0.17628 (0.0021)^2^
ICU mortality	-0.18046 (0.0017)^3^
Mortality at 6 months	-0.14882 (0.0097)^4^

## Discussion

In this retrospective cohort of comatose post-CA patients, adherence to the ERC-ESICM neuroprognostication guidelines was assessed and examined for changes following the 2021 guideline update [6]. This study provides three main insights: (1) implementation of these guidelines led to selective but meaningful improvements in specific prognostication practices, particularly biomarker use; (2) overall adherence, although slightly improved over time, remained moderate, with substantial heterogeneity across modalities; and (3) higher adherence was observed alongside with clinically relevant outcomes, particularly WLSM timing and mortality, underscoring the clinical implications of guideline-aligned prognostication.

One of the most notable facts associated with guideline implementation was the sharp increase in NSE measurement, which changed from being virtually absent before 2021 to becoming routinely integrated into multimodal prognostication thereafter. This finding is consistent with the 2021 ERC-ESICM guidelines, which elevated the role of biomarkers and emphasized repeated NSE measurements at 48 and 72 hours [6]. The marked rise in NSE values > 60 µg/L in the post-guideline period likely reflects improved systematic identification rather than a change in the underlying severity profile of patients. Similar improvements in biomarker use following guideline update have been observed in other studies [14-17].

On the contrary, adherence to other recommended modalities, including SSEP, EEG, neuroimaging, and timing of prognostication (≥72 hours) did not change significantly between periods. Several factors may explain this stability. First, baseline adherence to EEG and neuroimaging was already relatively high, limiting the potential for further improvement. Second, institutional and logistical constraints (availability of neurophysiology technicians and variability in expertise) may restrict real-world implementation of SSEP and NSE interpretation, which makes these methods continue to be less used when compared with EEG and neuroimaging methods. Interestingly, although EEG use did not increase, certain EEG patterns such as burst-suppression and electrographic seizures were more frequently documented in the post-guideline era [18].

The timing of prognostication is another core recommendation of the 2021 guidelines, which also did not significantly change in this study [6]. Timing of neuroprognostication and WLSM performed ≥72 hours after ROSC remained similar between the two periods. Previous multicenter studies have shown mixed adherence to timing recommendations, with delays often influenced by local resources, sedation practices, and workflow constraints. These findings align with work from Sandroni et al., who found that although most centers endorse the ≥72-hour rule, the real compliance is variable [19].

Clinical predictors such as myoclonus and fever at 72 hours, not dependent on diagnostic resources, remained unchanged, suggesting that changes were primarily driven by formal diagnostic modalities rather than bedside assessment. However, it is important to state that this assessment had been identified as markers of unfavorable neurological outcomes [20,21].

Longitudinal analysis demonstrated a gradual upward trend in adherence over the six-year period, with the most pronounced rise after 2021. This delayed response is consistent with broader literature describing the slow adoption of updated post-resuscitation guidelines. Multicenter analyses from the United Kingdom reported similar patterns, in which guideline recommendations take several years to be fully translated into clinical practice, influenced by training, resource availability, and institutional inertia [3,22]. Despite this progressive improvement in adherence, it remained modest, and the composite adherence score increased from only 3 to 4 points. Multimodal strategy appeared uneven across the multiple methods, while NSE use increased sharply, EEG and neuroimaging remained stable and high, and SSEP and timing-related parameters changed little. This heterogeneity reinforces the well-recognized difficulty in implementing complex multimodal prognostication practice and highlights the need for institution protocols and trained staff [23].

When approaching outcome associations, we should note that the negative correlation between adherence and survival reflects the clinical context in which prognostication is applied, rather than implying that greater adherence worsens outcomes. This may be explained by the fact that patients with poorer neurological trajectories were more likely to complete the full prognostication pathway, including modalities like SSEP and NSE, whereas rapidly recovering patients often require fewer or any prognostic tools. Sandroni and collaborators have previously shown that markers of poor prognosis, such as absent N20 responses or high NSE, drive the intensity of prognostication work-up, which may explain the association between adherence and mortality in our study. Nevertheless, the finding reinforces the importance of structured prognostication to avoid premature WLST-especially in settings where baseline mortality is high, and risks of self-fulfilling prophecies are substantial [12,24]. Regarding the association between adherence and clinical outcomes, higher adherence scores were significantly associated with WLSM ≥ 72 hours and overall WLSM, as well as mortality at hospital discharge and at six months [25,26]. The positive association between adherence and deferral of WLSM aligns with guideline intentions where higher adherence should favor postponement of prognostic decisions until after a complete multimodal assessment.

Despite the improvements in adherence reflected in a modest increase in the final adherence score, no significant differences were observed in ICU and six-month mortality. The lack of observed clinical impact may have multiple explanations. First, the improvements in adherence were limited to a few parameters, and the increase in the composite score was small. Second, patient characteristics and baseline severity were similar between groups, and improvements in adherence may not have been sufficient to influence outcomes in a severely ill population with high baseline mortality.

These findings highlight that implementing the 2021 ERC-ESICM guidelines improves specific elements of prognostication, particularly biomarker use, but does not automatically translate into widespread adoption of multimodal strategy [6]. The modest increase in overall adherence underscores persistent gaps between recommended practice and real use. These results suggest that guideline dissemination alone is insufficient, and structured educational programs, multidisciplinary protocols, and trained staff may be necessary to achieve consistent execution [27].

Furthermore, the association between adherence and WLSM timing demonstrates that structured prognostication can meaningfully influence ethically critical decisions [28]. Ensuring adherence may prevent premature WLSM in patients with potential for recovery, particularly in the early phase when neurological examination is unreliable, and, on the contrary, allows WLSM early when the neurological outcome is known to be poor, preventing family suffering [29,30].

Strengths and limitations

The study has several strengths, including a well-defined cohort, systematic evaluation of adherence across multimodal predictors, inclusion of temporal trends, and detailed assessment of associations with clinical outcomes. The composite adherence score represents an objective and replicable measure that may be useful for benchmarking practice across institutions.

Limitations must be acknowledged, including the retrospective design, potential unmeasured confounding, and the single-center setting, which may limit generalizability. Additionally, patients who regained consciousness within the first 24 hours were excluded, and their exact numbers were not systematically recorded; therefore, their potential impact on adherence estimates cannot be precisely quantified. Nevertheless, restricting analyses to patients with a motor score ≤ 3 at 24 hours reduces the likelihood that early awakeners substantially biased the overall adherence trends and helps mitigate this concern. Mortality associations must be interpreted cautiously due to indication bias, as sicker patients are more likely to undergo complete prognostication.

## Conclusions

Implementation of the 2021 ERC-ESICM neuroprognostication guidelines resulted in selective improvements in prognostication practices, particularly in biomarker measurement, and a modest overall increase in guideline adherence. Despite these changes, adherence remained heterogeneous, reflecting ongoing challenges in translating complex multimodal recommendations into routine clinical practice. Higher adherence was associated with more appropriate timing of WLSM, emphasizing the importance of guideline-aligned prognostication to support ethically sound decision-making. In fact, improved timing of neuroprognostication does not necessarily translate into reduced mortality, highlighting that adherence to guidelines primarily supports ethical decision-making rather than directly affecting survival. Future efforts should focus on structured implementation strategies to enhance consistent guideline uptake and reduce variation in neuroprognostication practices.
